# Burnout and Associated Factors among Hospital-Based Nurses in Northern Uganda: A Cross-Sectional Survey

**DOI:** 10.1155/2022/8231564

**Published:** 2022-03-24

**Authors:** Samson Udho, Amir Kabunga

**Affiliations:** ^1^Department of Nursing & Midwifery, Faculty of Health Sciences, Lira University, Uganda; ^2^Department of Psychiatry, Faculty of Health Sciences, Lira University, Uganda

## Abstract

**Background:**

Burnout is a public health problem that disproportionately affects nurses in sub-Saharan Africa because of the weak health systems that create an unconducive workplace environment. In Uganda, there is limited evidence on the burden of burnout among nurses in a manner that undermine advocacy and policy formulation. We aimed to assess the level of burnout and associated factors among nurses in northern Uganda.

**Methods:**

This was a cross-sectional survey conducted among 375 randomly selected nurses from health facilities in northern Uganda. Data were collected using a self-administered questionnaire. Data analysis consisted of descriptive statistics and logistic regression at a 95% level of significance in SPSS version 25.

**Results:**

Majority of the respondents were female 56.5% (*n* = 223). Nearly half, 49.1% (*n* = 194) of respondents had high levels of burnout, 36.2% (*n* = 143) reported average levels of burnout, and 14.7% (*n* = 58) reported low levels of burnout. Factors associated with burnout were age (AOR: 2.90; 95% CI: 1.28-6.58; *p* = 0.011), social support (AOR: 0.45; 95% CI: 0.22-0.94; *p* = 0.033), healthy eating (AOR: 0.06; 95% CI: 0.02-0.22; *p* < 0.001), workload (AOR: 0.31; 95% CI: 0.14-0.68; *p* = 0.004), and management responsibilities (AOR: 3.07; 95% CI: 1.54-6.12; *p* = 0.001).

**Conclusion:**

Half of the nurses in northern Uganda experienced high levels of burnout. The Ministry of Health should consider recruiting more nurses to reduce workload and adjust working hours to prevent workplace-related burnout among nurses in the country.

## 1. Introduction

Burnout is a public health problem with a pooled global prevalence of 15-60% among nurses [1]. Burnout is a phenomenon characterized by chronic stress, depersonalization, emotional exhaustion, and reduced work productivity, and the phenomenon may range from low to high levels [2]. Nurses in sub-Saharan Africa are disproportionately affected by burnout because of the weak health systems that create an unconducive workplace environment [3]. The prevalence of burnout among nurses in developed countries ranges from 49 to 57% while the burden of burnout in sub-Saharan African nurses range from 40 to 80% [4–7]. In Uganda, there is limited quantitative evidence on the burden of burnout among nurses; however, recent qualitative work showed evidence suggestive of nursing workforce burnout [8].

Nurses are the backbone of the health system workforce, and burnout among this cadre substantially undermines the quality of care offered to patients. Burnout among nurses has been associated with poor nurse-patient relationships, higher absenteeism, decreased empathy, substance abuse, depression, and suicidality [9–11]. Other effects include medical error, professional misconduct, low productivity, quitting the profession, and early retirement [12, 13]. However, burnout and its effects appear to differ across work settings and specialties [14]. Thus, more evidence is needed especially in low-income countries to allow policymakers to enact guidelines for protecting the nursing workforce.

Prior studies especially in high-income countries have examined the factors associated with burnout among nurses [15, 16]. These studies have demonstrated that the prevalence of burnout is linked majorly to personal factors like gender, age, marital status, having children, and working experience [17]. Organizational risk factors linked to burnout include work overload, low remunerations, and unsupportive peers and lack of control, practice setting, and career satisfaction [16–18]. However, the factors influencing burnout in nurses may vary from one setting to another and from one country to another. To inform workforce development efforts that will impact both current and future interventions to address burnout among nurses, this study was aimed at assessing the level of burnout and associated factors among nurses in the context of northern Uganda. We hypothesized that nurses working in northern Uganda where the healthcare system is generally poor experience a high level of burnout.

## 2. Materials and Methods

### 2.1. Design

This was a cross-sectional survey with quantitative methods of data collection conducted between February and March 2020. A cross-sectional study is where exposure and outcome are measured concurrently [19]. The reporting of this study followed the Strengthening the Reporting of Observational Studies in Epidemiology (STROBE) guidelines [20].

### 2.2. Study Setting

The study was conducted in two referrals, two general hospitals, and two health center levels IV (H/C IVs) in northern Uganda. These levels of health facilities were chosen because of the high number of nurses in the facilities. The two regional referral hospitals included Gulu and Lira Regional Referral Hospitals while the two general hospitals were Apac and Pentecostal Assembly of God (PAG) Mission Hospital. The H/C IVs included Aboke and Dokolo H/C IV. The two regional referral hospitals are found in Gulu and Lira City while the two general hospitals are located in Apac District and Lira City. The two H/C IVs are found in Kole and Dokolo District. The regional referral hospitals in Uganda provide comprehensive specialist services, conduct research, and training of health workers and serve about two million people [21]. The general hospitals on the other hand provide curative, preventive, maternity, outpatient, and inpatient services, and their target population are 500,000 people [21]. Meanwhile, the health center level IV provides curative, preventive, maternity, outpatient, and inpatient services, and their target population are 100,000 people [21]. The nurse-patient ratio in these facilities is estimated at 1: 11,000 [21].

### 2.3. Participants and Eligibility Criteria

The study included all nurses registered with the Uganda Nurses and Midwives Council (UNMC) working in the six health facilities as full-time permanent staff. Nurses who were busy and or attending to critically ill patients at the time of data collection were excluded from the study.

### 2.4. Sample Size and Sampling Procedure

The sample size was calculated using a single population proportion formula [1]. The z-score (*z*) was 1.96, the margin of error (*e*) was 0.05, and the prevalence (*p*) for the known population was 0.5 since the proportion of nurses with the prevalence of burnout in Uganda is not known. The calculated sample size was 427 plus a 10% allowance for nonresponse. Eligible participants were randomly enrolled in the study until the desired sample was realized.

### 2.5. Instrument

Stamm's Professional Quality of Life (ProQOL V-5) was used to assess the levels of burnout [22]. ProQOL V-5 has 30 items measuring compassion fatigue, satisfaction, and burnout but only ten items measuring burnout were analyzed. The burnout subscale has an alpha scale reliability of 0.75, and interscale correlations indicate 5% shared variance (*r* = −0.14; co − *σ* = 2%; *n* = 1187) [22]. The ProQOL was preferred because it has been used in Uganda and found to be valid and reliable [23]. The ProQOL V-5 is a 5-point Likert scale ranging from 1 to 5. A burnout score of ≤22 indicates low levels, 23 to 41 denotes average levels, and ≥42 signifies high levels of burnout. The Cronbach's *α* coefficient of burnout was 0.82. We also developed a set of demographic and contextual questions contributing to burnout to form the independent variables. The independent variables were both demographic (age, gender, marital status, level of education, and working experience) and contextual factors (workplace bullying, economic status, workload, health eating, enough sleep, management responsibility, spiritual support, and social support). The respondents were asked to respond to a “yes” or “no” question on workplace bullying, social support, spiritual support, healthy eating, sleep, and management responsibility. Additionally, they were asked whether their current economic status was satisfactory or not and asked to state their working hours in a typical working day.

### 2.6. Data Collection Procedure

We used a self-administered questionnaire to collect data from the nurses across the six health facilities. The nurses who volunteered to participate in the study were given a questionnaire to fill during their free time. Completion of the questionnaire was estimated to take on average 20 minutes. The research assistants then collected the questionnaires the following day.

### 2.7. Data Analysis

Data were analyzed using SPSS version 23.0. A data entry screen was created in SPSS version 23 with checks to ensure accuracy during entry. Data were scanned for out-of-range and missing values before commencing data analysis. Continuous variables were summarized as median, mean, and standard deviations, while categorical variables will be summarized as frequencies and percentages. The level of burnout (outcome variable) was recategorized as a binary of low and high burnout based on scores below and above the mean score. A univariate logistic regression was performed to test the association between the outcome variable and the independent variables. Variables *p* value < 0.05 were included in a multivariate logistic regression model to establish the factors independently associated with burnout. The analysis was conducted at a 95% level of significance, and the corresponding adjusted odds ratio (AOR) and *p* value were reported.

### 2.8. Ethical Considerations

This study was approved by an accredited research and ethics committee based in a university (university's name and approval number redacted). Participants in this study were recruited based on written informed consent after explaining the purpose, risks, and benefits of participating in the study. Confidentiality was maintained throughout the research process. The study was anonymous, and the participants had the right to withdraw at any time with no penalty.

## 3. Results

Out of the 427 calculated sample size, only 409 responded to the questionnaire giving a response rate of 95.8%. However, 12 questionnaires were excluded due to missing information on burnout giving a total of 395 participants for the final analysis.

### 3.1. Demographic Characteristics of Study Participants (*N* = 395)

A total of 395 participants were included in the analysis, and their characteristics are presented in Table 1. The results indicate that 56.5% (*n* = 223) were female, 53.7% (*n* = 212) were aged 30 years and above, 54.2% (*n* = 214) had education level of above a diploma, 51.9% (*n* = 190) were married, and 52.2% (*n* = 206) had worked for over six years.

### 3.2. Level of Burnout among Nurses in Northern Uganda (*N* = 395)

Based on the ProQOL manual guidelines, 49.1% (*n* = 194) had high levels of burnout (≥42 score), 36.2% (*n* = 143) reported average levels of burnout (23-41 score), and 14.7% (*n* = 58) reported low levels of burnout (≤22 score).

### 3.3. Workplace Contextual Factors Associated with Burnout among Study Participants (*N* = 395)

The majority of the participants experienced workplace bullying (50.6%), lacked social support (54.2%), and considered their economic status to be satisfactory (53.2%). Conversely, the minority of the participants worked for less than 10 hours per shift (48.9%) and had management responsibility (49.4%) (Table 2).

### 3.4. Factors Associated with Burnout among Nurses in Northern Uganda (*N* = 395)

During the bivariate logistic regression analysis (Tables 1 and 2), the factors associated with burnout among the study participants were age (*p* < 0.001), work experience (*p* < 0.001), workplace bullying (*p* = 0.005), economic status (*p* = 0.002), social support (*p* < 0.001), spiritual support (*p* = 0.002), healthy eating (*p* < 0.001), having enough sleep (*p* = 0.002), workload (*p* < 0.001), and management responsibilities (*p* < 0.001). After controlling for confounding in the multivariate logistic regression, the factors that were independently associated with burnout among the study participants were age (AOR: 2.90; 95% CI: 1.28-6.58; *p* = 0.011), social support (AOR: 0.45; 95% CI: 0.22-0.94; *p* = 0.033), healthy eating (AOR: 0.06; 95% CI: 0.02-0.22; *p* < 0.001), workload (AOR: 0.31; 95% CI: 0.14-0.68; *p* = 0.004), and management responsibilities (AOR: 3.07; 95% CI: 1.54-6.12; *p* = 0.001) (Table 3).

## 4. Discussion

Our study was aimed at assessing the level of burnout and associated factors among hospital-based nurses in northern Uganda. We found that close to 50% (*n* = 194) of the nurses had high levels of burnout, and the factors independently associated with burnout were age, social support, healthy eating, workload, and management responsibilities. These results suggest that burnout is a major public health problem among nurses in Uganda with many contributing factors that need to be addressed.

The high level of burnout (49.1%) observed in this study may be justified by the weak health systems of Uganda characterized by a shortage of nurses and the contextual workplace factors identified in this study [24]. The results of this study are comparable to the 49% reported in America, 50% in Jordan, and 48% in Egypt [7, 25, 26]. Compared to previous studies, our findings reported higher levels of burnout than 19% reported in America and 18.3% in Brazil [16, 27]. Relatedly, the 49.1% level of burnout observed in our study is much higher than 16.0% reported in China, 35.7% in Portugal, and 31.0% in Italy [28–30]. However, our results were lower than 53.6% observed in the Brazilian state of Bahia, 50.8% in Sao Paulo, 55.4% in the state of Sao Paulo, and 56.9% in Greece [4, 5, 31, 32]. This difference could be attributed to the differences in health systems across countries and the composition of the sample where some studies involved all healthcare workers including doctors, nurses, midwives, and other cadres [6]. These findings demonstrate the importance of promoting a supportive workplace environment among nurses to seek help when needed while recognizing burnout and its effects.

Our findings showed that participants who were < 30 years of age were three times more likely to experience burnout compared to their counterparts who were > 30 years of age. This relationship seems to be in line with the observation that burnout may reduce as professionals get older. This may be attributed to the development of better coping strategies by older and experienced nurses as old age is associated with professional experience [15, 33]. Our results are in agreement with previous research which shows a correlation between burnout and age [34]. However, contrary to our findings, other studies did not find a relationship between burnout and age [35, 36]. The disparity in findings may be attributed to differences in measurements of burnout and the timing of conduct of the study. For instance, the study by Batayneh et al. [35] used the Maslach Burnout Inventory (MBI) while we used the Stamm's Professional Quality of Life (ProQOL V-5) to measure burnout. Relatedly, unlike our study, the study by Kabunga and Okalo [36] was conducted in the context of Coronavirus disease 2019 (COVID-19). Nonetheless, our results suggest a need to devise mechanisms to protect younger nurses against workplace-related factors such as heavy workload that predispose them to burnout.

Our study also revealed that nurses who had social support had 55% fewer odds of experiencing burnout compared to their colleagues who had no social support. Our findings suggest that inadequate perceived social support can lead to increased burnout. Social support is a relevant resource that determines the quality of life and wellbeing of nurses [37]. Substantial research has indicated that social support can ameliorate the negative impact of burnout [38]. As predicted in our study, studies show that social support is associated with low levels of burnout among health care professionals [38]. Therefore, providing social support to the nurses may help the nurses to cope effectively with burnout [39]. Consequently, several studies underscore the need for establishing programs to give healthcare professionals social support as an essential component [40].

Interestingly, we observed that participants who had healthy eating habits had 94% fewer odds of experiencing burnout compared to their colleagues who did not have healthy eating habits. Although our findings do not prove a causal-effect relationship, it is tempting to speculate a possible pathway to burnout among nurses. Research evidence shows that a healthy diet influences the prevalence of mental disorders [40]. While the exact mechanism is still debatable, it is assumed that low-grade inflammation leads to pathogens of the disease [40]. A healthy diet has an anti-inflammatory impact on the body [40]. Therefore, healthy eating may have effects on burnout in nurses and recent studies have indicated that healthy eating may have protective effects against burnout [41].

The results of the present study indicated that nurses who worked for less than 10 hours a day were 31% less likely to experience burnout compared to their colleagues who worked for more than 10 hours a day. This may not be a surprising result given the shortage of nursing workforce in the country. The total number of health professionals required in Uganda is 167,765. In 2019, however, the number stood at just 27,612 [42]. Generally, health professionals in the sub-Saharan African region have a heavy workload owed to shortages in the health workforce caused by brain drain and nursing care is the most affected. Studies show that long working hours inhibit the opportunities for recovery necessary to be healthy [43]. Our findings suggest that working for long hours has detrimental effects on nurses through fatigue, health, and pleasure deprivation which is supported by other studies [44]. In line with our findings, studies in Iran and Sweden showed that long working hours are associated with burnout [45–47]. Our results suggest a need to rethink the duration of work shifts for nurses to prevent burnout.

Lastly, our results revealed that nurses who had extra management responsibilities were three times more likely to experience burnout compared to their colleagues who did not have any management responsibilities. This was an expected result because, in addition to providing nursing care, nurses with management functions are faced with several relational tasks including organization, motivation, conflict resolution, and meetings. Thus, management responsibilities lead to a heavy workload and this is a major risk factor for burnout [46]. Given the fact that a highly stressful environment leads to burnout, extra load in form of management responsibilities may increase susceptibility to burnout [48]. This result is similar to Jennings' study who found a relationship between nurse managers and burnout. According to this study, the demanding role of nurse managers results in burnout [49]. Likewise, in a qualitative study of nurses in England, nurse managers reported high levels of burnout [50]. Thus, to preserve the mental welfare of nurse managers, all possible efforts should be made to minimize burnout. Possible interventions may include senior officials lending their support and helping reduce managers' responsibilities by providing more supplementary staff [51].

## 5. Limitations

The study is limited geographically because it was conducted in two referral and four general hospitals in northern Uganda. Therefore, the results may not be generalized to all nurses in Uganda. Secondly, excluding nurses who were busy and or attending to critically ill patients at the time of data collection could have introduced selection bias and thus underestimation of the prevalence of burnout among hospital-based nurses. Finally, due to the cross-sectional nature of the study, the causality between variables cannot be established. Nonetheless, the current study potentially expands previous research suggesting that burnout is associated with workplace-related factors.

## 6. Conclusion

Burnout among nurses in northern Uganda is high with 1 in 2 of the nurses experiencing high levels of burnout. Burnout is associated with age, social support, healthy eating, workload, and management responsibilities. The findings of this study give insights into what should be done to improve the workplace environment of nurses in Uganda to prevent workplace-related burnout. The Ministry of Health should consider interventions such as recruiting more nurses to reduce workload, adjusting working hours, and ensuring hours of effective rest to prevent workplace-related burnout. More research is needed to understand how workplace-related burnout among nurses impacts job performance, satisfaction, and intention to quit a job in the context of Uganda.

## Figures and Tables

**Table 1:** A univariate logistic regression of demographic characteristics associated with burnout among study participants (*N* = 395).

Variables	Category	Frequency	Percent	*p* value
Gender				0.668
	Male	172	43.5	
	Female	223	56.5	
Age				<0.001^∗^
	<30 years	183	46.3	
	>30 years	212	53.7	
Education levels				0.725
	<diploma	181	45.8	
	>diploma	214	54.2	
Marital status				0.194
	Single	205	48.1	
	Married	190	51.9	
Work experience				<0.001^∗^
	<6 years	189	47.8	
	>6 years	206	52.2	

^∗^Statistically significant variables at *p* < 0.05.

**Table 2:** A univariate logistic regression of workplace contextual factors associated with burnout among study participants (*N* = 395).

Variables	Category	Frequency	Percent	*p* value
Health facility level				0.262
	Regional referral	158	40	
	General hospital	134	33.9	
	Health center IV	103	26.1	
Workplace bullying				0.005^∗^
	Yes	200	50.6	
	No	195	49.4	
Economic status				0.002^∗^
	Satisfactory	210	53.2	
	Less satisfactory	185	46.8	
Social support				<0.001^∗^
	Yes	214	54.2	
	No	181	45.8	
Spiritual support				0.002^∗^
	Yes	214	54.2	
	No	181	45.8	
Healthy eating				<0.001^∗^
	Yes	243	61.5	
	No	152	38.5	
Enough sleep				0.002^∗^
	Yes	210	53.2	
	No	185	46.8	
Workload				<0.001^∗^
	<10 hours	193	48.9	
	>10 hours	202	51.1	
Management responsibilities				<0.001^∗^
	Yes	195	49.4	
	No	200	50.6	

^∗^Statistically significant variables at *p* < 0.05.

**Table 3:** A multivariate logistic regression of factors associated with burnout among nurses in northern Uganda (*N* = 395).

		Burnout levels				
Variable	Category	Low = 75	High = 320	COR 95% CI	AOR 95% CI	*p* value
Age						
	<30 years	11 (6.0)	172 (90.0)	6.76 (3.44-13.33)	2.90 (1.28-6.58)	0.011^∗^
	>30 years	64 (30.2)	178 (69.8)	1.00	1.00	
Economic status						
	Satisfactory	52 (24.8)	158 (75.2)	0.43 (0.25-0.74)	2.01 (0.94-4.30)	0.071
	Less satisfactory	23 (12.4)	162 (87.6)	1.00	1.00	
Social support						
	Yes	58 (27.1)	156 (72.9)	0.28 (0.16-0.50)	0.45 (0.22-0.94)	0.033^∗^
	No	17 (9.4)	164 (90.6)	1.00	1.00	
Healthy eating						
	Yes	72 (29.6)	171 (70.4)	0.05 (0.02-0.16)	0.06 (0.02-0.22)	<0.001^∗^
	No	3 (2.0)	149 (98.0)	1.00	1.00	
Workload						
	<10 hours	64 (33.2)	129 (66.8)	0.12 (0.06-1.3)	0.31 (0.14-0.68)	0.004^∗^
	>10 hours	11 (5.4)	191 (94.6)	1.00	1.00	
Management responsibilities						
	Yes	15 (7.7)	180 (92.3)	5.14 (2.80-9.44)	3.07 (1.54-6.12)	<0.001^∗^
	No	60 (30.0)	140 (70.0)	1.00	1.00	

^∗^Statistically significant variables at *p* < 0.05; COR: crude odds ratio; AOR: adjusted odds ratio; 1.00: reference category.

## Data Availability

The datasets used and/or analyzed during the study are available from the corresponding author on reasonable request.
